# Fabrication of Silica Nanospheres Coated Membranes: towards the Effective Separation of Oil-in-Water Emulsion in Extremely Acidic and Concentrated Salty Environments

**DOI:** 10.1038/srep32540

**Published:** 2016-09-06

**Authors:** Yuning Chen, Na Liu, Yingze Cao, Xin Lin, Liangxin Xu, Weifeng Zhang, Yen Wei, Lin Feng

**Affiliations:** 1 Department of Chemistry, Tsinghua University, city, 100084, P. R. China

## Abstract

A superhydrophilic and underwater superoleophobic surface is fabricated by simply coating silica nanospheres onto a glass fiber membrane through a sol-gel process. Such membrane has a complex framework with micro and nano structures covering and presents a high efficiency (more than 98%) of oil-in-water emulsion separation under harsh environments including strong acidic and concentrated salty conditions. This membrane also possesses outstanding stability since no obvious decline in efficiency is observed after different kinds of oil-in-water emulsions separation, which provides it candidate for comprehensive applicability.

Materials for oil-water emulsion separation have aroused great interest due to their extensive application in industrial area^1^,^2^,^3^,^4^,^5^,^6^,^7^. Large number of oily wastewater is daily produced because of the sharp development of chemical industry and manufacturing leading to formidable threats to environment as well as the livelihood of people. Traditional treatment of the wastewater including floatation, flocculation treatment, precipitation method and so forth are low efficient and high energy consumed. Recently, as the oil-water separation is essentially an interfacial issue, a novel strategy that utilizes special wettability of materials to separate oil-water mixtures is demonstrated to be efficient and low-cost^8^,^9^,^10^,^11^,^12^,^13^,^14^,^15^,^16^. Materials are rendered superoleophilic-superhydrophobic and superhydrophilic-underwater superoleophobic to achieve oil-water separation, which are systematically referred to as “oil-removing”^17^,^18^,^19^,^20^,^21^,^22^,^23^ and “water-removing”^24^,^25^,^26^,^27^,^28^ materials.

Except the separation of simple oil-water mixture, materials have been purposively designed for application in specific environments since wastewater is highly emulsified or corrosive in most cases inevitably. On one hand, it is worth noting that oily waste water is frequently discharged in the form of oil-in-water emulsions, because of various surface-active substance. The surfactants form a stable interfacial layer between oil and water phases, which invalidates traditional oil-water separating membranes. To solve this challenge, free-standing films and functionalized filter membranes with tiny apertures or channels have been processed to separate emulsions^29^,^30^,^31^,^32^,^33^,^34^,^35^,^36^. On the other hand, not only mild environments but also corrosive conditions appear in the oil-water separation process. For instance, concentrated sulfuric acid, as a kind of common inorganic acid, is widely used in petroleum refining and chemical production as catalyst or reactant resulting in extreme acidification of wastewater. Besides, frequent oil spill accidents require materials with tolerance to concentrated salty conditions. Up to now, materials such as chitosan, nitrocellulose, and silica gel, have been selected in fabricating separation membrane resulting from their outstanding corrosion resistance^37^,^38^,^39^. However, as far as we concerned, there are few membranes can separate oil-in-water emulsion under strong acidic and concentrated salty conditions simultaneously, which is of great significance in industrial applications and remains great challenge that becomes a huge drive behind the recent development of oily wastewater purification technology.

Herein, a superhydrophilic-underwater superoleophobic membrane is fabricated by coating silica spheres onto a glass fiber membrane through a sol-gel process. Glass fiber membranes are pre-treated by ammonia water-hydrogen peroxide mixed solution and 1,2-Bis(triethoxysilyl)ethane (BTSE) solution to produce roughened and silanized substrates. The silica coated membranes, fabricated with substrates which are successively undergoing two pre-treatments, own a complex framework with micro-nano structure covering and present the highest efficiency (more than 98%) of oil-in-water emulsion separation under harsh environments including strong acidic and concentrated salty conditions. As it working on different kinds of oil-in-water emulsions, no obvious decline in efficiency is observed indicates that our membranes possess outstanding stability and comprehensive applicability.

## Results

**Figure 1:**
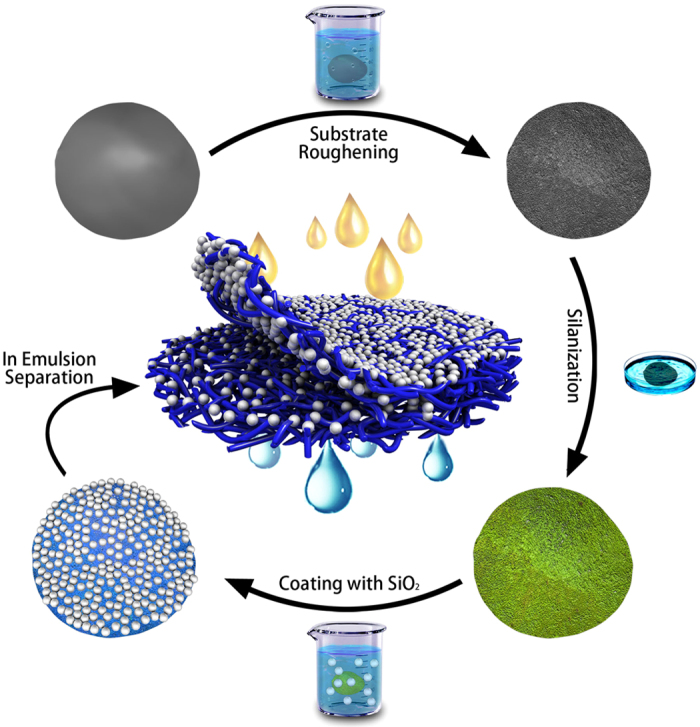
Schematic illustration of membrane fabrication and emulsion separation. Firstly, glass fiber membrane is roughened by immersion in ammonia water-hydrogen peroxide mixed solution. Secondly, the membrane is transferred in BTSE solution for silanization. Then, the pre-treated substrate is gradually coated with SiO_2_ spheres during a sol-gel process. Finally, the membrane for emulsion separation in harsh conditions is obtained after rinsing with ethanol and water. During emulsion separation, oil-in-water emulsions are demulsified when contacting and infiltrating the membrane. Oily phase is obstructed by the membrane due to underwater superoleophobicity of the membranes. Meanwhile, water phase smoothly penetrates the membrane on account of its superhydrophilicity. (The authors are grateful for copyright authorization of the image used in Fig. 1 from Beijing MyScimage Multimedia Technology Center).

Figure 1 is a schematic illustration of the membrane fabrication and emulsion separation. Firstly, glass fiber membrane, which is used as substrate with average pore size of 220 nm, is immersed in ammonia water-hydrogen peroxide mixed solution at 60 °C for 30 min. During this process, the substrate becomes fluffy resulting from the formation of numerous bubbles. Secondly, the fluffy substrate is transferred in BTSE solution for silanization which fractional Si-O-Si bond forms by hydrolysis^40^,^41^. Then, the pre-treated substrate is immersed in an aqueous solution consisting of Tetraethoxysilane (TEOS) and ammonia water and gradually coated with SiO_2_ spheres generated through a slow sol-gel process. Finally, the membrane for emulsion separation in harsh conditions is obtained after rinsing with ethanol and water for removal of reactants and redundant SiO_2_ spheres. The as-prepared membrane has a complex framework combining layers which own different types of micro-nanostructures and leading to demulsification toward oil-in-water emulsions in strong acidic and concentrated salty conditions as well as gentle environments. The oil-in-water emulsions are demulsified when contacting and infiltrating the membrane as shown in center of Fig. 1. Oily dispersion phase coalesce and is repelled by the membrane due to underwater superoleophobicity of our membranes. In contrast, water as the continuous phase smoothly penetrates the membrane on account of its superhydrophilicity. The whole process is only driven by gravity without any further consumption of energy.

**Figure 2:**
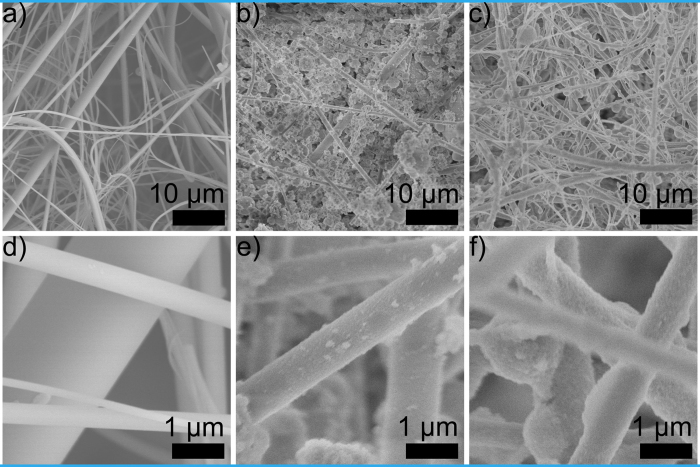
SEM images of membranes. (**a**) SEM image of initial glass fiber membrane. (**b**) SEM image of final fabricated membrane. (**c**) SEM image of interlayer of the membrane whose surface layer has been peeled off. (**d–f**) Corresponding high-resolution SEM images to (**a–c**) orderly.

Scanning electron microscope (SEM) images of membranes are shown in Fig. 2. The glass fiber membrane which we used as initial substrate is shown in Fig. 2a,d with a higher resolution. Glass fibers owning smooth surfaces random stack forming membrane filters with irregular voids of which average pore size is about 220 nm. To our surprise, the fabricated membrane has a unique hybrid structure which is inferred to be profoundly influenced by the pre-treatments on substrates. Large numbers of bubbles generate resulting in many cavities inside the final product and a higher roughness in micro-scale on its surface. In macro perspective, the as-prepared membrane is thicker, fluffier and can be peeled off layer by layer facilely, which can not achieve by the initial substrates. SEM images of the as-prepared membrane whose surface layer is peeled off are shown in Fig. 2c,f. It is unexpected that fibers are spindle-shaped with numerous nano-papillae covering rather than cylindrical. SEM images in perspective of cross-section are given in Figure S1, in which cavities and cracks are clearly visible. As reported before, the micro-nanostructure can enhance the wettability. The hydrophilic materials will present superhydrophilic when they own a rough surface which is composed of a large amount of micro-nano structures. Therefore, the complex structure combining layers which own different types of micro-nanostructure can endow the prepared membrane with special wettability^27^,^42^,^43^.

**Figure 3:**
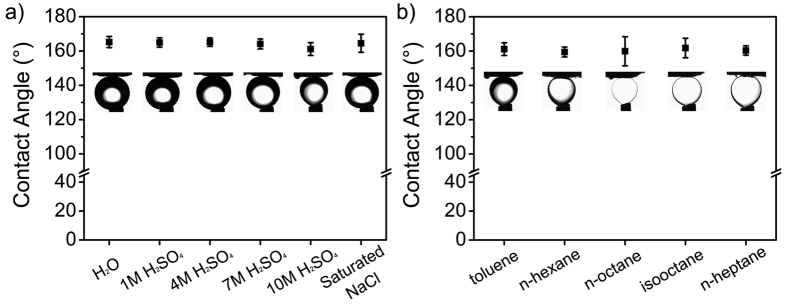
Oil contact angles of the as-prepared membrane under water phase. (**a**) Oil contact angles of the membrane measured under a series of H_2_SO_4_ solutions in gradient concentration and saturated sodium chloride solution when toluene is chosen as the oil phase. (**b**) Oil contact angles of the membrane measured in 10M H_2_SO_4_ solution, oil phase ranges among toluene, n-hexane, n-octane, isooctane and n-heptane.

Specific combination of wettability, such as superhydrophilicity and underwater superoleophobicity, is the foundation for oil-water separation. To characterize the wetting behavior of our membrane, water contact angles (WCAs) and oil contact angle (OCAs) were measured in air and under water respectively. As shown in Figure S2, WCA of the membrane is about 0° showing superhydrophilic property obviously. To characterize underwater superoleophobicity, oil droplets were extruded in controlled volume from a needle and driven to contact the surface of membrane from the below side under the water phase, since density of oil phase is relatively lower than water phase. As shown in Fig. 3, OCAs of the membrane were measured under a series of aqueous sulfuric acid (H_2_SO_4_) in gradient concentration and saturated sodium chloride solution when toluene was chosen as the oil phase (Fig. 3a). Because of the micro-nanostructure on the surface and the corrosion resistance of SiO_2_, OCAs are over 160° in not only strong acidic but also concentrated salty conditions demonstrating stable underwater superoleophobicity of our membranes. When oil phase changes, such as toluene, n-hexane, n-octane, isooctane, n-heptane, OCAs still remain nearly 160° under 10 M H_2_SO_4_ aqueous solutions resulting in broad applicability of our membranes. Wettability measurements prove that our membranes are underwater superoleophobic, stable in harsh environment and available for repellence against various oils. These properties offer the possibility for emulsion separation in strong acidic and concentrated salty environments.

Besides special wettability and corrosion resistance, the ability to separate emulsions under harsh conditions is highly controlled by the micro-nanostructures and corresponding nano-channels of the membrane^44^. To highlight the effect of the micro-nanostructures, three kinds of membranes were fabricated to separate emulsions for comparison. The as-prepared membrane that has been roughened and silanized in two pre-treatments mentioned above is referred to as **RSM** (**R**oughened and **S**ilanized **M**embrane). The membrane that was fabricated as RSM without roughening in ammonia water-hydrogen peroxide mixed solution is referred to as **NSM** (**S**ilanized **M**embrane with No roughening). In other words, the initial glass fiber membrane was directly silanized, then functionalized by the following sol-gel process, and finally rinsed by ethanol and water. Similarly, **NNM** is defined as the membrane prepared without any pre-treatments. In other words, initial substrate was immediately immersed in TOES-ammonia water mixed aqueous solution and coated with SiO_2_ spheres generated through the slow sol-gel process. Then the SiO_2_ spheres-coated substrate was rinsed with ethanol and water to obtain NNM. The differences in morphology and efficiency of emulsion separation in harsh conditions among three kinds of membranes were characterized and discussed in details as follows.

SEM images of NSM and NNM are shown in Figure S3a,b orderly, and Figure S3c,d are corresponding SEM images in high resolution. The average pore size of both membranes decline accordantly as RSM. However, the morphology of three membranes is quite different. NNM presents a hybrid of glass fibers and SiO_2_ spheres which both haphazard stack and lack mutual adhesion, which is caused by lacking of silanization step in manufacturing process. BTSE, used as one kind of cross-linker, leads to SiO_2_ spheres uniformly coating the fibers instead of forming isolated spheres. Especially on NSM (Figure S3a,c), the majority of SiO_2_ forms a layer coating around fibers with nano-roughness on its surface where spheres of SiO_2_ mostly disappear. It is worth noting that neither NSM nor NNM can be peeled off layer by layer facilely like RSM, which demonstrates that the substrate roughening step is the key to the formation of framework of RSM.

**Figure 4:**
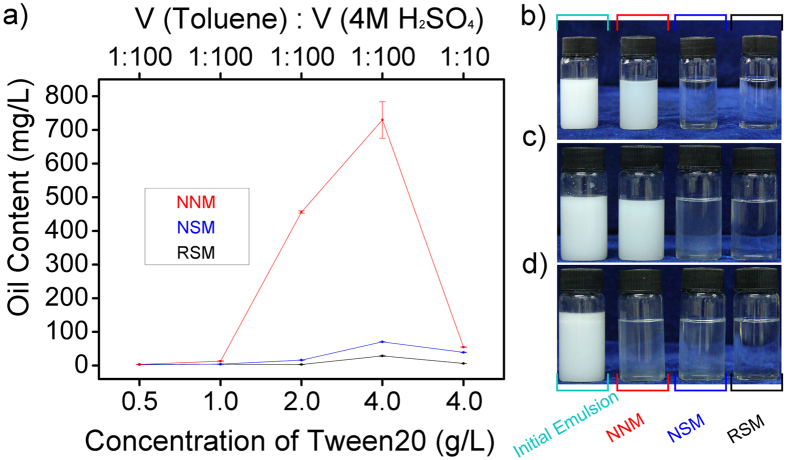
Emulsion separation utilizing RSM, NSM, NNM. (**a**) Oil content in filtrate collected from emulsion separation in which RSM, NSM, NNM have been used. Emulsions have been prepared with toluene and 4M H_2_SO_4_ in volume ratio of 1:100 or 1:10, and gradient concentration of Tween-20 are separately added as surfactant. (**b–d**) Photograph of initial emulsion and filtrate collected from emulsion separation in which RSM, NSM, NNM have been used. (**b**) Emulsions have been prepared with toluene and 4M H_2_SO_4_ in volume ratio of 1:100, and 2.0 g L^−1^ Tween-20 is added. (**c**) Emulsions have been prepared with toluene and 4M H_2_SO_4_ in volume ratio of 1:100, and 4.0 g L^−1^ Tween-20 is added. (**d**) Emulsions have been prepared with toluene and 4M H_2_SO_4_ in volume ratio of 1:10, and 4.0 g L^−1^ Tween-20 is added.

As shown in Fig. 4, three kinds of membranes were used to separate different kinds of emulsions mixed with toluene and 4 M H_2_SO_4_ in volume ratio of 1:100 and 1:10. Gradient concentration of Tween-20 were separately added as surfactant, and oil-in-water emulsions were obtained after vigorous stirring for 90 min. In emulsion separation experiments, 30 mL as-prepared emulsion was poured onto the membrane which was fixed and supported by a pair of glass fixture, then demulsified during permeating the membrane. Water phase penetrated through the membrane directly, while oil phase was repelled and gradually coalesced by reason of superhydrophilicity and underwater superoleophobicity which were introduced by natural property of SiO_2_ and micro-nano composite structure of membranes. The filtrate was collected in vials and photographed together with initial emulsions (Fig. 4b–d). Besides, the concentration of residual oil in filtrate was measured to evaluate the ability of three membranes for emulsions separation in harsh conditions.

As shown in Fig. 4a, three membranes present similarly when a relatively small quantity of surfactant exists. The concentration of residual oil remains at a low level and the filtrate appears transparent. The distinction in ability for emulsions separation appears more clearly accompanied with the increasing concentration of surfactant. RSM maintains the efficiency of emulsion separation which is a little bit better than NSM, while NNM presents the worst performance. NNM loses its ability for emulsion separation and the filtrate remains creamy white after filtration. This phenomenon is consistent with the results manifested in photos (Fig. 4b–d). This result indicates that the complex structure combining layers which own different types of micro-nanostructure ensures the stable ability of RSM for emulsion separation in harsh conditions.

**Figure 5:**
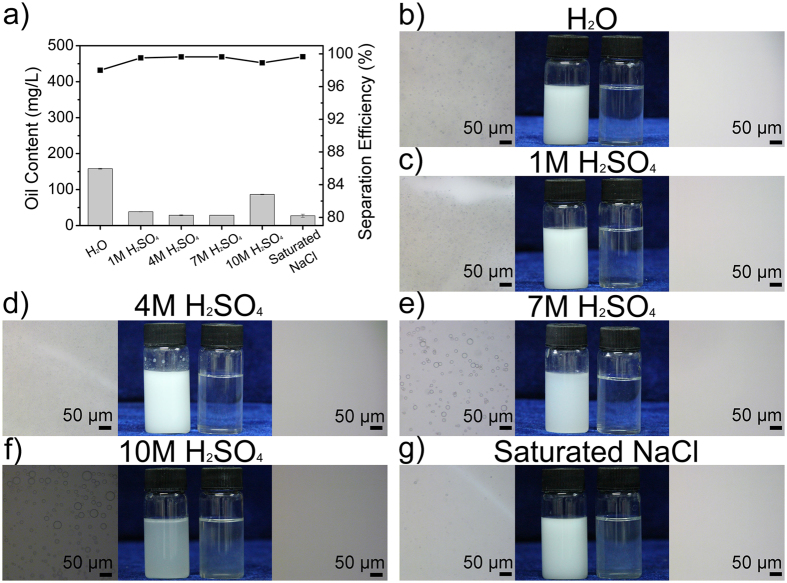
Emulsion separation using SiO_2_ coated membrane to investigate its resistance against strong acidic and concentrated salty conditions. (**a**) Residual oil content in filtrate and corresponding separation efficiency. Toluene was selected as the oil phase, deionized water, H_2_SO_4_ aqueous solution in gradient concentration, and saturated NaCl solution were chosen as water phases separately. All of these emulsions were mixed by toluene and water phase in volume ratio of 1:100 and surfactant-stabilized on account of addition of 4.0 g L^−1^ Tween-20. (**b–g**) Optical microscope images of initial emulsion, photograph of initial emulsion and filtrate, optical microscope images of filtrate from left to right in each experiment.

Because of its best performance, RSM was chosen to separate multifarious oil-in-water emulsions for investigating its resistance against strong acidic and concentrated salty conditions. Separation efficiency was also calculated in this experiment. Similar to the wettability measurement, toluene was selected as the oil phase, deionized water, H_2_SO_4_ aqueous solution in gradient concentration, and saturated NaCl solution were chosen as water phases separately. All of these emulsions were mixed by toluene and water phase in volume ratio of 1:100 and surfactant-stabilized on account of addition of 4.0 g L^−1^ Tween-20. It is the maximum dosage we used in comparison experiment mentioned above. NNM loses its ability of emulsion separation in this case, though it used to be extremely effective in separating emulsions with lower dosage. As shown in Fig. 5, oil contents in filtrate remain at a low level ranging from 30 to 150 mg L^−1^. Correspondingly, the phenomenon which each emulsion separation efficiency is more than 98% indicates that the membrane has outstanding resistance to harsh conditions. Data shown in Fig. 5b–g is highly consistent with this result. Filtrates are transparent contrasted with the creamy white initial emulsions. Oil droplets disappear in the optical microscope images of filtrates (on the right side) instead of comprehensive distribution in initial emulsions (on the left side). It is obvious that the efficiency in separating emulsion made with deionized water is relatively lower than others. We infer that this phenomenon is caused by the change of ion concentration. High concentration of ions can weaken emulsification by weakening the hydration of surfactant, which makes emulsions a little bit unstable. Therefore, separation efficiency increases in separating emulsions with high ion concentration on the premise that membranes would not be destroyed by the harsh environments.

**Figure 6:**
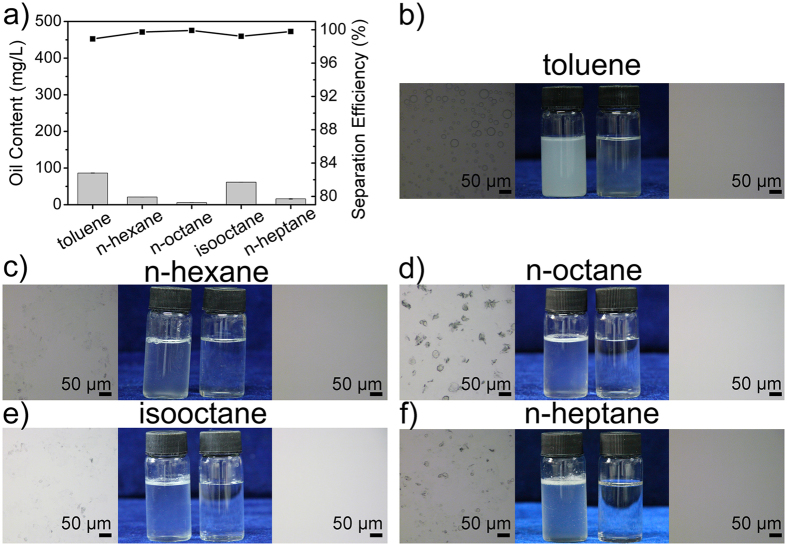
Emulsion separation using the as-prepared membrane to investigate its applicability. (**a**) Residual oil content in filtrate and corresponding separation efficiency. Toluene, n-hexane, n-octane, isooctane, and n-heptane are selected as the oil phase, separately. 10M H_2_SO_4_ aqueous solution is chosen as water phases. All of these emulsions are mixed by toluene and water phase in volume ratio of 1:100 and surfactant-stabilized on account of addition of 4.0 g L^−1^ Tween-20. (**b–f**) Optical microscope images of initial emulsion, photograph of initial emulsion and filtrate, optical microscope images of filtrate from left to right in each experiment.

In consideration of practical application, the as-prepared membrane was used to separate emulsions prepared with different kinds of oils, such as toluene, n-hexane, n-octane, isooctane, and n-heptane (Fig. 6). 10 M H_2_SO_4_ was selected as water phase. Volume ratio between oil phase and water phase remained 1:100, and 4.0 g L^−1^ Tween-20 was added as surfactant. As we expected, the membrane successfully separates five kinds of emulsions with few residual oil left. Efficiencies are more than 99% corresponding to data in photos as well as the images of optical microscope (displayed similarly with Fig 5). Additionally, the separation of mixture-emulsions was tested. Emulsions prepared from mixing two/three kinds of emulsions, which were used to calculate separation efficiency mentioned earlier, were separated (Figure S4). To our surprise, three kinds of mixture-emulsions have been separated successfully and efficiencies are more than 99%. For supplement, the experiment in which emulsion was suction filtered under low pressure environment by a water pump was executed (Figure S5). The relative vacuum degree was about −0.1 MPa. Emulsion was prepared with toluene and 10M H_2_SO_4_ in volume ratio of 1:100, and 4.0 g L^−1^ Tween-20 was added. The separation efficiency slightly decreases since some dispersive oil droplets in nano-scale are forced to permeate the membrane instead of sufficient aggregation and coalescence when approaching the underwater superoleophobic membranes. However, the separation efficiency remains acceptable about 96.76% demonstrating that our membrane has comprehensive applicability.

## Discussion

In summary, a superhydrophilic-underwater superoleophobicmembrane is fabricated for oil-in-water emulsion separation in strong acidic and concentrated salty conditions. The preparation method has been optimized. The resulting membrane has tolerance against strong acidic and concentrated salty environment keeping superhydrophilic-underwater superoleophobic stably. Consequently, the membrane can separate oil-in-water emulsions with addition of surfactant in harsh conditions, and separation efficiencies are more than 98%. Additionally, various oil-in-water emulsions made with different kinds of oil can be separated by this membrane.

## Methods

### Materials

The glass fiber membrane was commercial and the product specification is marked by the manufacturers with a diameter of 50 mm and average pore size of 220 nm. The commercial membrane was directly used without any additional clipping. TEOS was purchased from J&K Scientific Ltd. CCl4 was purchased from Tianjin Aoran Fine Chemical Research Institute. BTSE was purchased from Nanjing Capatue Chemical Co. Ltd. Other reagents (such as toluene, n-hexane, n-octane, isooctane, n-heptane, etc.) were purchased from Beijing Chemical Co. Ltd. Deionized water was used.

### Instrumentation and Characterization

The SEM images of as-prepared meshes were obtained using a field-emission scanning electron microscope (SU-8010, Hitachi, Japan). Contact angles were measured on a contact angle measurement machine (OCA15 machine, Data-Physics, Germany) at ambient temperature. The oil concentration in filtrate was measured using an infrared spectrometer oil content analyzer (Oil480, Beijing Chinainvent Instrument Tech. Co. Ltd., China). CCl4 was used to extract oil from filtrate. Optical microscopy images were taken on a polarizing microscope (Nikon ECLIPSE LV100POL, Japan).

### Fabrication of Membrane

Firstly, glass fiber membrane used as substrate was immersed in ammonia water-hydrogen peroxide mixed solution at 60 °C for 30 min (H_2_O_2_: NH_3_H_2_O: H_2_O = 1: 1: 5 v/v). Secondly, the membrane rinsed by deionized water was transferred in BTSE solution (5% vol) for 20 min. Thirdly, the pre-treated substrate is immersed in TEOS solution stirring for 24 h. TEOS solution was prepared as follows: 10.4g TEOS was dissolved in 350 mL ethanol under vigorous stirring for 30 min, then 5 mL H_2_O and 55 mL NH_3_H_2_O were added in solution. Finally, the as-prepared membrane was rinsed with ethanol and water for removal of reactants and redundant SiO_2_ spheres.

### Fabrication of Emulsions

Oil phase (toluene, n-hexane, n-octane, isooctane and n-heptane) and water phase (deionized water, 1M, 4M, 7M, 10M H_2_SO_4_ aqueous solution and saturated NaCl aqueous solution) were mixed in conical flasks at the ratio of 1:100 or 1:10 (v/v) with addition of surfactant (0.5, 1.0, 2.0, 4.0 g L^−1^ Tween-20). The emulsions were obtained after vigorous stirring for 90 min.

### Emulsion Separation Experiments

The membrane was fixed between a pair of glass fixture which was attached with glass tubes. The fixture supported the membrane which was used as a filtration cell during separation process. 30 mL as-prepared emulsion was poured onto the membrane and demulsified during permeating the membrane. Water phase penetrated through the membrane directly, while oil phase was blocked above. The filtrate was collected in vials under fixture. Oil content in filtrate was measured to calculate separation efficiency according to


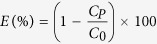


where E(%) is the separation efficiency. C_*P*_ and C_0_ are the oil content of the filtrate and the initial emulsion, respectively.

## Additional Information

**How to cite this article**: Chen, Y. *et al*. Fabrication of Silica Nanospheres Coated Membranes: towards the Effective Separation of Oil-in-Water Emulsion in Extremely Acidic and Concentrated Salty Environments. *Sci. Rep.* **6**, 32540; doi: 10.1038/srep32540 (2016).

## Supplementary Material

Supplementary Information
