# Supplementary material for: Fabrication of Silica Nanospheres Coated Membranes: towards the Effective Separation of Oil-in-Water Emulsion in Extremely Acidic and Concentrated Salty Environments
Source: Sci Rep. 2016 Sep 6;6:32540. doi: 10.1038/srep32540 (PMC5011648; doi:10.1038/srep32540)
Supplement: Supplementary Information [file srep32540-s1.pdf]

Electronic Supplementary Information

Fabrication of Silica Nanospheres Coated Membranes: towards the Effective Separation of Oil-in-Water Emulsion in Extremely Acidic and Concentrated Salty Environments

Yuning Chen, Na Liu, Yingze Cao, Xin Lin, Liangxin Xu, Weifeng Zhang, Yen Wei, and Lin Feng*

Department of Chemistry, Tsinghua University, Beijing 100084, P. R. China.
E-mail: fl@mail.tsinghua.edu.cn; Tel: +86-010-62792698

This Supplementary Information section contains:

1. SEM images in perspective of cross-section.
2. Water contact angle of membrane in air.
3. SEM images of NSM and NNM.
4. Separation of mixture-emulsions.
5. Separation under pressure environment.

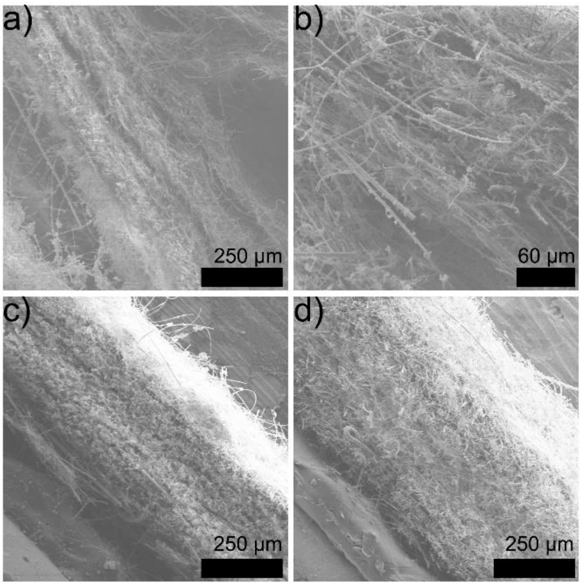

Fig. S1 SEM images in perspective of cross-section. a) Cross-sectional SEM images of RSM. b) Cross-sectional SEM images of RSM in high resolution. c) Cross-sectional SEM images of NSM. d) Cross-sectional SEM images of NNM.

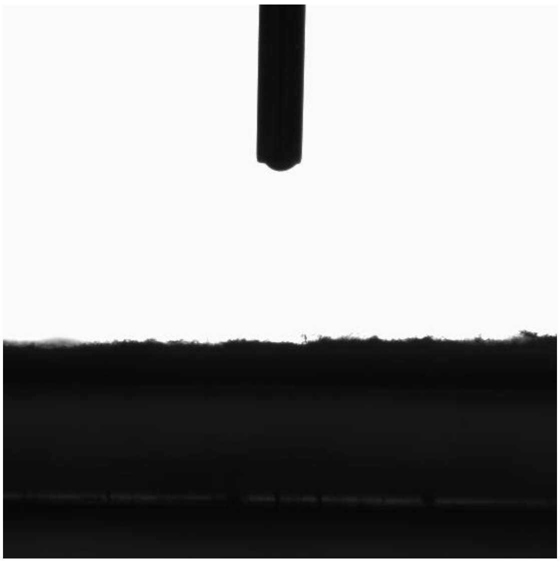

Fig. S2 Water contact angle of membrane in air.

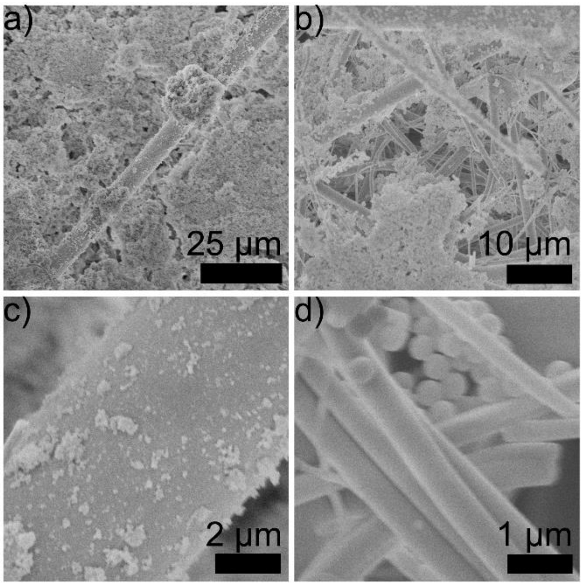

Fig. S3 SEM images of NSM and NNM. a) SEM images of NSM. b) SEM images of NNM. c) SEM images of NSM in high resolution. d) SEM images of NNM in high resolution.

In consideration of practical application, separation of mixture-emulsions has been tested. The emulsions prepared from mixing two/three kinds of emulsions, which have been used to calculate separation efficiency in our manuscript, have been separated. To our surprise, three kinds of mixture-emulsions have been separated successfully and efficiencies are more than 99%.

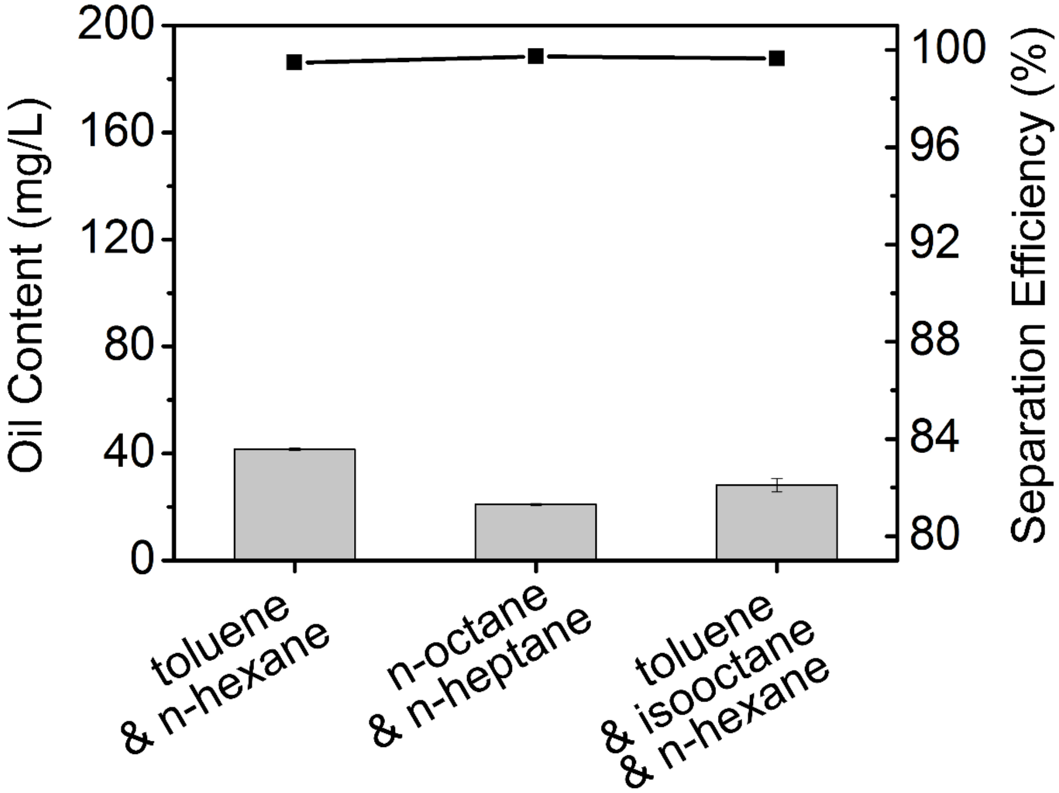

Fig. S4 Residual oil content in filtrate and corresponding separation efficiency.

An experiment in which emulsion is suction filtered under low pressure environment by a water pump is executed. The relative vacuum degree is about -0.1 MPa. Emulsion has been prepared with toluene and 10M H_2SO_4 in volume ratio of 1:100, and 4.0 g L^{-1} Tween-20 is added. The separation efficiency slightly decreased since some dispersive oil droplets in nano-scale are forced to permeate the membrane instead of sufficient aggregation and coalescence when approaching the underwater superoleophobic membranes. However, the separation efficiency remains acceptable about 96.76%.

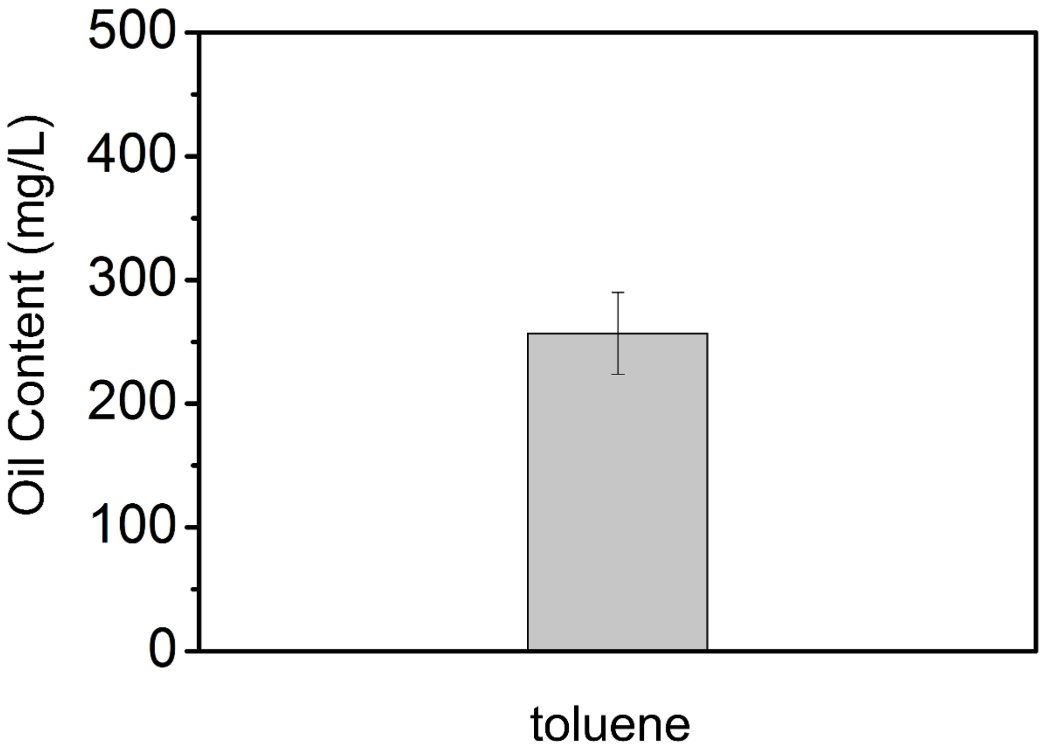

Fig. S5 Residual oil content in filtrate collected in suction filtration.
